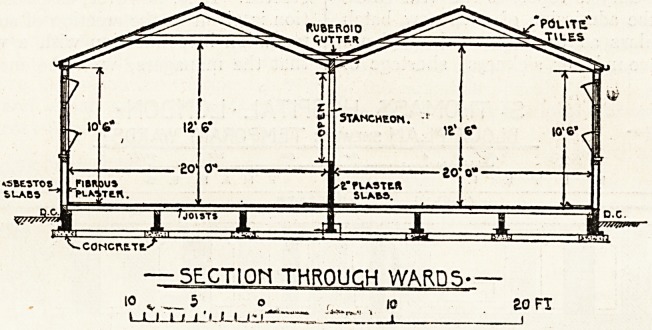# The New Huts at St. Thomas's Hospital: The Problem of Additional Accommodation

**Published:** 1915-06-12

**Authors:** 


### THE VOLUNTARY HOSPITALS AND THE WOUNDED.

### The New Huts at St. Thomas's Hospital.

### THE PROBLEM OF ADDITIONAL ACCOMMODATION.

One of the great difficulties which might have arisen from the reception of wounded sailors and soldiers into the voluntary hospitals is due to the necessity of having the patients under discipline, and the enforcement of War Office routine.

#### Need for Military Superintendents.

Unless some special provision has been made for the purely military work connected with the wounded, trouble and overwork for the superintendent or secretary of the hospital and his department must result. The best plan is to have an officer appointed as the military superintendent, who effectually buffers off from the secretary's office all the purely military work connected with the soldier-patients. It is his duty to report to the War Office on the day after the admission of each new batch of patients particulars as to regimental numbers, the condition of wounds or sickness, shortage of clothing, and other matters. All inquiries relating to soldiers are referred to the military superintendent's office, and all regulations as to discharge, conveyance to relief hospitals, and arrangements for furlough are in the hands of the military superintendent. The appointment is, of course, an honorary one, and is best carried out by one of the consulting surgeons to the voluntary hospital, as has proved so successful at the London Hospital, where Colonel Hurry Fenwick holds the appointment of military superintendent.

#### The Limit of Beds for Soldiers.

Another important point is one we dealt with last week. As the demand for accommodation for ever-increasing numbers of wounded has developed, the War Office has naturally endeavoured in the first instance to obtain as much accommodation as possible for such cases in the voluntary hospitals. When a hospital, after consultation with its medical staff, has fixed the number of beds which can be devoted to the reception of sick and wounded soldiers, without injustice to the civil population, it is of the first importance that the managers should cry a halt. The voluntary hospitals have rendered magnificent service to the military authorities, but they cannot do impossibilities, and the example of the London Hospital should everywhere be followed by hospital managers declining to attempt to increase the number of beds placed at the service of the War Office beyond the maximum which they find it possible to work with the greatest efficiency.

#### Spare Ground and Temporary Wards.

There remains the question of vacant space represented by a garden or grass plots or a vacant site in part of the hospital grounds on which temporary pavilions for the reception of the wounded might be erected. It is, however, essential, before any sanction is given to the erection of such further accommodation in connection with a voluntary hospital, that the managers, with the medical staff, should satisfy themselves that they are not attempting to supply more beds than they can properly administer and equip. This limitation has been reached at the London Hospital, where the committee have had the courage to decline to erect huts on the limited open spaces within the hospital area on the ground that they cannot provide the staff and equipment to administer such additional wards with efficiency and success.

At St. Thomas's Hospital the authorities have been fortunate enough to attract offers of service from a large number of old St. Thomas's men and of old nurses and sisters, so that the committee have felt justified in erecting some excellent huts for the reception of 300 wounded. The way in which the site necessary for their accommodation has been utilised will be readily seen from the accompanying plan of the hospital, showing the new hut pavilions in the interspaces between the main buildings. We made an inspection of these huts and found them amongst the best buildings of the kind to be met with anywhere. So excellent are they that they might well form a new unit for this class of construction. We commend the study of them to every hospital and every institution, and also to local authorities who find themselves under the necessity to erect temporary hospital buildings for the reception of patients.

Details of the Huts.

The sketch-plan on the previous page shows the details of the size and arrangements of the huts. They contain 300 beds; the actual cost of the structure has been £6,600, and when the cost of all fittings, including lavatories, etc., is added, the total amounts to £10,040. In other words, it has cost under £34 per bed to provide this excellent accommodation. These huts, like those of the famous Moabit Hospital in Berlin, though of a temporary character, are capable of doing good service for a lengthened period. This once more illustrates the direction in which future hospital construction should develop. The days of hospital buildings costing from £700 to £1,000 per bed ought speedily to be relegated to the limbo of forgotten things.

Gifts from the Governors and Staff.

The public spirit exhibited by the Governors of St. Thomas's Hospital and the noble support they have received from the men and women who owe their skill and knowledge in medicine, surgery, and nursing to the great parent hospital are worthy of the gratitude and support of the public. The Governors of St. Thomas's Hospital badly need help in this matter to defray the expenses, which have been very heavy, and also to enable them to supplement the equipment, which is in many respects good, but somewhat incomplete as compared with the usual hospital standard. The Government have paid the cost of the structure, fittings, etc., but outside help will be very highly appreciated by the Governors, and we hope that help will be forthcoming in liberal measure. The wives of members of the staff of St. Thomas's have established a fund to supply creature comforts, including periodicals, writing materials, cigarettes, games, etc., a sum of £300 has been already received, and further donations will be welcome if sent to Mr. G. Q. Roberts, the secretary of St. Thomas's Hospital.

The following is a sectional plan of the new war-huts at St. Thomas's Hospital. The brief technical description of the structure which is appended cannot fail to prove interesting to many readers.

Technical Details of Construction.

The wards are constructed with timber framing on brickwork foundations taken just above level of ground. The framework is covered on the outer face with "Polite" slabs (composed of asbestos, wood, and Portland cement). The inside walls are covered with fibrous plasters, slabs, and distempered. The roofs are constructed with 4-in. by 2-in. rafters and ties, and covered with "Polite" tiles upon 2½-in. battens. The gutters are covered with "Ruberoid" in two thicknesses.

The partitions dividing the back-to-back wards are constructed with 4-in. by 2-in. stancheons, fixed from floor to roof, about 10 or 12 ft. apart, and the space between is filled up to a height of 5 ft. 3 in. from the floor with 2-in. plaster slabs. The space above this is open to allow of free circulation of air.

The floors are constructed with 6-in. by 2-in. joists on sleeper walls, and covered with 1-in. grooved and tongued floor boards. The space under is ventilated by iron gratings.

All walls have felt damp-proof courses. The windows are placed 6 ft. from centre to centre, and the height is divided into three lights. The bottom and top lights are hinged to fall inwards into hoppers. The wards are heated by gas radiators. The sanitary offices are drained into existing drains.

—SECTION THROUGH WARDS.—

## Figures and Tables

**Figure f1:**
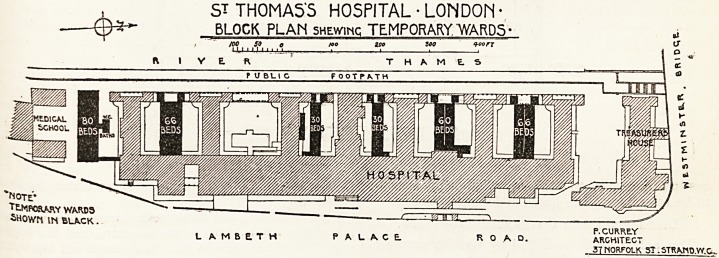


**Figure f2:**